# Monthly Summary

**Published:** 1894-09

**Authors:** 


### Monthly Summary.

A CASE IN PRACTICE.—Some time ago I was called to the house of a lady who had been suffering from toothache for some time. When I arrived the lady told me that Dr. ——— had treated her for a length of time but could not relieve her of her pain, and finally told her that she was not susceptible to medicine, and that she would have to consult a specialist. Upon examination I found that the lady had a copper amalgam filling in the first upper molar, and first and second bicuspids badly decayed. I extracted the molar at once and found the roots abscessed, with a large sac on one of them. I expected that this would relieve her of her pain, but the following day she came to my office and complained of having the same pain. I now treated the bicuspids and dismissed her to come again at a certain time. The second following day she came with the same complaint. I removed the dressing from the bicuspids and devitalized the pulps, extracted them, and prepared the canals with an antiseptic dressing, intending to fill them the following day. (This was not done in one sitting; it took several.) When she came she complained that the pain was worse than ever. Now I was in a dilemma, and did not know what to do. I again examined her teeth very carefully and found every one apparently sound. I happened to remember a case I had many years ago, where all the teeth in the patient's mouth were sound, but that one molar had a peculiar sound different from the rest when tapping it. I extracted this molar and found, after splitting it, that the nerve was dead and the odor from it was anything but pleasant. I now determined to drill into the second molar, and as soon as I reached the pulp chamber with the drill a quantity of pus was discharged and the pain was immediately gone. I treated the canals and in due course of time filled them as well as the canals in both bicuspids, and —quietness reigns supreme. This case shows that in apparently sound and healthy teeth diseased pulps may be present. *Monatsschrift Deutscher Zahnkunstler.*

HOT WATER FOR HEMORRHAGE.—Dr. Julius Scheft, Jr., of Vienna, according to the current number of *Ash's Quarterly Circular*, recommends strongly the use of hot water for arresting hemorrhage after tooth extraction. "We are accustomed," he writes, "to stop hemorrhage by the method that has been used for generations, viz., by the direct application of cold water to the wound. Practitioners started with the idea that heat caused expansion of and induced increased bleeding from the vessels; but on the other hand, cold caused contraction. In an ordinary case of extraction, hemorrhage from the arteria dentalis, or from the gums and periosteum, soon ceases; but it frequently happens, even when the patient does not suffer from hematophila, that there is difficulty in arresting the flow of blood." Dr. Scheft mentions three cases occurring in his practice, in each of which there is a history of profuse hemorrhage after extraction. "I allowed one patient," he says, "to take a great quantity of cold water, and yet there appeared not the slightest diminution in the bleeding. I then took a glass syringe and continuously applied hot water, in drops, to the wound, from which the blood previously trickled without cessation. After a few seconds the bleeding diminished, a coagulum was formed, and the bleeding finally ceased. With the second patient I used hot water at once, and the flow of blood was arrested. In the third case the wound had been bleeding for a long time. I plugged the alveolus with iodoform gauze, and on removing the plug the wound bled afresh. I then employed hot water; the hemorrhage ceased and did not recur." Dr. Scheff applies the hot water by means of a syringe, injecting it by drops into the socket of the tooth. The arrest of hemorrhage in surgical operations by the application of heat is a recognized resource, and it would therefore seem that this principle might with advantage be applied in cases of tooth extraction, especially as the mouth is able to bear a very high temperature without inconvenience. In fact, water so hot that it causes pain when the finger is inserted in it will in many cases be tolerated in the mouth.—*Lancet.*

AMBLYOPIA CAUSED BY DENTAL IRRITATION.—Patient complained of headache, weakness and seeing specks before her eyes, and, a few days later, of a weakness of the sight of the right eye. Her vision was so impaired that she could scarcely count her fingers at a distance of ten inches from the eyes. The cause could not be discerned by an opthalmoscopic examination. She was treated with iodide of potassium for optic neuritis, but her vision became dimmer. A physician suggested to examine her teeth and found five ground off roots on the right side, upon which a vulcanite plate rested. The roots were extracted, and four days later her vision was better. In less than two months her eyesight was completely restored and remained so, as subsequent examination showed. —*Dominion Dental Journal.*

---

BLEACHING WITH PYROZONE.—At the recent joint meeting of the Maryland State and Washington City Dental Societies, Dr. C. A. Meeker, of Newark, N. J., successfully bleached a deeply discolored left superior central incisor in thirty minutes. His method, after carefully adjusting the rubber dam, is to wipe out the prepared cavity with ammonia, this to neutralize possible acidity; then with a gold probe, armed with a swab of bibulous paper saturated with pyrozone, 25 per cent. he liberally moistens the interior of the cavity and exterior surface of the tooth, evaporating the solution by repeated blasts of cold air. This method is continued till the tooth shines out again in its original color. The doctor is highly elated over his success with pyrozone as a bleaching agent, and speaks encouragingly of the stability of its results.—*Items of Interest.*

---

CLEANING THE TEETH.—A two per cent aqueous solution of trichloracetic acid to moisten the pumice is perfectly harmless. I have used it in my practice for some time, and find it far superior to tincture iodin for removing the "green stain" on children's teeth.—*W. H. Jones, N. Y.*

THE USE OF PEROXIDE OF HYDROGEN AS A HÆMOSTATIC.—In the *New York Medical Journal*, Dr. Brewer recommends peroxide of hydrogen for the purpose of stopping hemorrhage. He has found it of great value in a number of cases. He states that it has been thought by some that the hæmostraction of this drug is due in great measure to the fact that the peroxide-of-hydrogen fluid when brought into contact with organic matter is rapidly decomposed, giving off free nascent oxygen. This by uniting with the hæmoglobin, converts the blood, which is generally venous in character and charged with carbon dioxide, into bright arterial blood, which it is well known, is much more readily coagulable than the vitiated fluid.

Rewer was, however, led to question the correctness of this theory when he observed that in some of his most alarming cases the bleeding was distinctly arterial in character, and that these cases seemed to yield to the treatment as readily as those in which the oozing was undoubtedly venous.

Dr. W. G. Thompson placed the writer in possession of some facts which he observed during a series of experiments with this agent.

He observed that when a strong solution of peroxide of hydrogen (fifteen volumes or more) was brought into contact with the blood by being directly injected into the veins during life, it caused a rapid disintegration of the red corpuscles, resulting in the immediate precipitation of a semi-solid detritus which completely occluded the smaller vessels: that this effect extended so rapidly throughout the vascular system that within a comparatively short space of time the animal operated on would present the appearance characteristic of the most profound anæmia.

Thompson also observed that the application of strong solutions of this agent to mucous and serous membranes caused an almost immediate degeneration of the epithelial cells, resembling at first a rapid and exaggerated cloudly swelling, and later a complete destruction of the tissue.

In view of these facts, the writer is inclined to believe that the arrest of hemorrhage which is observed when strong peroxide of hydrogen is applied to a freshly-wounded surface results from this remarkable disintegrating action both on the tissues and the blood, rather than the simple oxygenation and rapid coagulation of the latter, for it is easy to understand how the rapid swelling of the endothelial lining of the cut blood-vessels and the solidification of the blood would result in a more or less complete obliteration of their lumen.—*Ther. Gazette.*

---

KEEPING THE GINGIVAL PORTION OF A CAVITY DRY.—I have found the use of trichloracetic acid preferable to carbolic acid, the eschar which it makes being much dryer than that formed by carbolic acid. It can be used of the same strength as prepared for use in the treatment of "pyorrhea," viz., by opening the original ounce bottle of crystals and simply filling the inter-spaces with water, making an ounce bottle of liquid trichloracetic acid from the entire original bottle of crystals. The application of trichloracetic acid of this strength keeps the gum margin so dry that oxiphosphate can be inserted in contact with it without injury.

I find the matrix a valuable adjunct in the use of amalgam in many cases, using for this purpose a thin piece of German silver or cold-rolled steel, trimmed to proximate the curve of the cervical margin and forced just a trifle between the gum and the margin of the cavity. The labio-lingual plane of the matrix can be formed with crowning-pliers to any shape desired; in some cases perfectly straight, and in others even convex at the gingival portion of the surface next the cavity. A matrix thus formed can be held in place with a wedge or two, having the wedges made by the assistant and kept on hand in various sizes, preferably with scarcely any taper from end to end, but with the coronal edge thinner than the gingival edge, this difference being easily increased by the insertion of a smaller wedge at the side of the larger one near the gingival edge. It is scarcely necessary to add that a wedge is never used a second time. The matrix may also be secured by tying a thread around it and the tooth. A matrix thus made and secured consumes but little time and facilitates the work very much, and also avoids lacerating the gums by the use of steel or orange-wood to smooth the cervical margin. The matrix being held in close proximity to the cervical margin, it is not necessary to use any instruments on the surface of that portion of the filling, and the "knuckle" has a smooth surface, which more nearly proximates the adjoining tooth than in shaping it with instruments.

The adaptation of the matrix to the surface of the root of the tooth can be made more perfect (especially does this apply to the superior bicuspids) by allowing the thread to contain a knot or two against the center of the matrix.—*Cosmos.*

---

GOLD IN PAUPER'S TEETH SAVED.—Nothing is useless nowadays—not even a defunct pauper. Hood's plaintiff wail:

> Rattle his bones
> Over the stones;
> He's only a pauper.
> Whom nobody owns—

is now out of date. The guardians know better than to act so recklessly, for often the party concerned is the possessor of a set of artificial teeth which contains a good deal of gold—last relict of more prosperous times—and in the interest of the rate payers the precious metal must be secured and turned into pounds, shillings and pence. Often, too, they are the owners of rings or tiny trinkets, not pawnable, but still containing as much auriferous value as not a few modern gold mines. These have to be collected and also converted into cash by means of the melting pot. In the Holborn Union the melting process takes place once a year, and has just been accomplished for the present season. The jewelry dealt with is what is found on paupers who die friendless and unclaimed in its various establishments. Rings, chains, brooches and trinkets have been melted down, and produced a bar of gold, estimated at eleven carat, and worth about £40. A good proportion of it was got from the plates of artificial teeth. Mr. Walton said that on one set of teeth there was at least £4 worth of gold. The proceeds are paid into the common exchequer of the union.—*London Telegraph.*

TO SECURE RUBBER DAM.—A year or so ago Dr. Hetrick gave a hint as to the value of sandarach varnish to secure rubber-dam to the teeth, instead of the painful silk ligature. It was one of the most valuable items I ever received, and if it has not been universally adopted it should be. It is absolutely demanded that dentistry should be made as easy as possible, and any neglect is scarcely less than criminal.

Many patients in a country practice are so situated that a prolonged course of treatment for aching or ulcerated teeth is out of the question. When operating for such an one, and an exposed living nerve is found, it can almost always be successfully removed at once by injecting a solution of cocain, under strong pressure, so that it is forced up to the apex of the roots, otherwise the removal will not be painless. When a fragment of these fresh pulps resists all efforts of the broach to effect removal, the heating of the Evans root-canal dryer and passing up into the root brings the fragment away at once. The potassium and sodium compound of Dr. Schrier will also disintegrate one of these fresh pulps so it can be taken out at once.—*Dr. Bergstresser.*

---

TREATMENT OF EPITHELIOMA.—Darier (*La Medicine Moderne*, May 12, 1894) has obtained excellent results with the following dressing: First dry the ulcerated surface, then cauterize with any cautery,—thermo-cautery, or, still better, chromic acid,—and finally use a solution of 1 to 100 of methyl-blue, either in repeated paintings or in interstitial injections. Recovery often follows at the end of one or two months. Abadie, from personal observation, believes that the chromic acid is unnecessary, and that the methyl-blue alone is effective. He points out that the improvement is often exceedingly slow, and months may pass without appreciable change, and this probably accounts for the fact that it has been abandoned by many practitioners.—*The Therapeutic Gazette.*

